# Low-Energy Laser-Driven Ultrashort Pulsed Electron Beam Irradiation-Induced Immune Response in Rats

**DOI:** 10.3390/ijms222111525

**Published:** 2021-10-26

**Authors:** Gohar Tsakanova, Nelly Babayan, Elena Karalova, Lina Hakobyan, Liana Abroyan, Aida Avetisyan, Hranush Avagyan, Sona Hakobyan, Arpine Poghosyan, Bagrat Baghdasaryan, Elina Arakelova, Violetta Ayvazyan, Lusine Matevosyan, Arpine Navasardyan, Hakob Davtyan, Lilit Apresyan, Arsham Yeremyan, Rouben Aroutiounian, Andreyan N. Osipov, Bagrat Grigoryan, Zaven Karalyan

**Affiliations:** 1 Institute of Molecular Biology NAS RA, Yerevan 0014, Armenia; babayanelly@gmail.com (N.B.); karalovae@gmail.com (E.K.); lina.hakobyan@gmail.com (L.H.); pbzhikyan@yahoo.gr (L.A.); a.avetis@mail.ru (A.A.); a.avagian@yahoo.com (H.A.); 777sona7@gmail.com (S.H.); arpi.poghosyan21@gmail.com (A.P.); bagdasaryanbagrat@gmail.com (B.B.); elinaa72@mail.ru (E.A.); viola_ay@yahoo.com (V.A.); lusine.matevosyan@inbox.ru (L.M.); lilit000apresyan@gmail.com (L.A.); rouben_a@hotmail.com (R.A.); zkaralyan@yahoo.com (Z.K.); 2 CANDLE Synchrotron Research Institute, Yerevan 0040, Armenia; arpinenavasardyan@gmail.com (A.N.); davtyan@asls.candle.am (H.D.); arsham.yeremyan@gmail.com (A.Y.); grigory@asls.candle.am (B.G.); 3 Department of Genetics and Cytology, Faculty of Biology, Yerevan State University, Yerevan 0025, Armenia; 4 Experimental Laboratory, Yerevan State Medical University after Mkhitar Heratsi, Yerevan 0025, Armenia; 5 Group for Radiation Biochemistry of Nucleic Acids, N.N. Semenov Federal Research for Chemical Physics, Russian Academy of Sciences, 119991 Moscow, Russia; andreyan.osipov@gmail.com; 6 Laboratory for the Development of Innovative Drugs and Agricultural Biotechnology, Moscow Institute of Physics and Technology, 141701 Moscow, Russia; 7 Experimental Radiobiology and Radiation Medicine Department, State Research Center—Burnasyan Federal Medical Biophysical Center of Federal Medical Biological Agency, 123098 Moscow, Russia

**Keywords:** laser-driven ultrashort pulsed electron beam, *Wistar* rats, whole-body irradiation, peripheral blood profiling, bone marrow profiling, lymph node profiling, proinflammatory cytokines, DNA damage and repair, spleen, thymus

## Abstract

The development of new laser-driven electron linear accelerators, providing unique ultrashort pulsed electron beams (UPEBs) with low repetition rates, opens new opportunities for radiotherapy and new fronts for radiobiological research in general. Considering the growing interest in the application of UPEBs in radiation biology and medicine, the aim of this study was to reveal the changes in immune system in response to low-energy laser-driven UPEB whole-body irradiation in rodents. Forty male albino *Wistar* rats were exposed to laser-driven UPEB irradiation, after which different immunological parameters were studied on the 1st, 3rd, 7th, 14th, and 28th day after irradiation. According to the results, this type of irradiation induces alterations in the rat immune system, particularly by increasing the production of pro- and anti-inflammatory cytokines and elevating the DNA damage rate. Moreover, such an immune response reaches its maximal levels on the third day after laser-driven UPEB whole-body irradiation, showing partial recovery on subsequent days with a total recovery on the 28th day. The results of this study provide valuable insight into the effect of laser-driven UPEB whole-body irradiation on the immune system of the animals and support further animal experiments on the role of this novel type of irradiation.

## 1. Introduction

The application of electron beams in radiobiomedicine and the commercial development of medical electron linear accelerators goes back to the early 1950s, when a huge amount of research in the field of radiation therapy conducted by different leading centers provided reliable evidence of the benefits and advantages of this kind of irradiation for the treatment of certain types of cancer [1]. Although electron beams are most widely used for the treatment of superficial tumors (<5 cm deep), they were shown to be on a level with superficial X-ray treatment and even better for larger tumors [2], including skin and lip cancers, breast cancer, node irradiation, and head and neck cancers [3], successfully providing the uniform dose distribution in the target volume and mitigating the surrounding healthy tissue from radiation damage [2,3].

However, new laser-driven electron linear accelerators are now available, providing unique ultrashort pulsed electron beams (UPEBs) with ultrashort picosecond pulses of low repetition rates of just several Hertz, but with doses several orders of magnitude higher (10^11^ Gy/min) than those provided by conventional beams [4,5,6,7,8,9]. It is assumed that accelerators of this type, by delivering ultrashort sub-picosecond electron pulses, tremendously reduce the interaction time, preventing the site chemical effects in biological samples, and thus opening effective outlooks for radiation therapy. It was shown that very-high-energy electron beams allow higher radiation doses to be delivered in deep tissue, thereby providing potential clinical benefits for targeting deep tumors and improving the tumor-to-healthy-tissue ratio by delivering a higher radiation dose to the tumor versus normal tissues [10,11]. These features allow this new modality to be considered in future long-term biomedical studies as a new, promising, more effective, and less harmful radiotherapeutic approach for cancer treatment.

The investigations on the biological effect/effectiveness of conventional, not laser-driven, accelerators have been conducted for several decades [12,13,14,15,16,17,18]. However, since ultrashort pulsed laser-driven electron accelerators are relatively new, data about their radiobiological effect is very limited in the literature, and the existing studies are mostly related to in vitro studies [19,20,21,22,23,24]. Thus, laser-driven UPEB irradiation has been shown to induce molecular-cytogenetically visible copy number variations (CNVs) [19], increase micronucleus frequency, and shorten telomere length in healthy human peripheral blood lymphocytes [18,25]. In the human ovarian cancer cell line *OVCAR-3*, it was shown to reduce the cell survival [25]. The immediate and reversible DNA damage by a single femtosecond irradiation shot at 1 Gy with a very high dose rate of 10^13^ Gy/s per pulse was observed in the A431 human skin carcinoma cell line [7]. In human fibroblasts, UPEB radiation produces more complex DNA damage than X-ray radiation, leading to cell death rather than cytogenetic disturbance [20].

The investigation of the formation of these effects is only at the initial stage; in fact, it is a new and promising direction in radiation biology and medicine. Although the immune system is on the first line among the other systems and pathways undergoing radiation toxicosis, in the literature there are no reports on the studies of the adaptive post-irradiation reactions of the immune system in response to irradiation with a low-energy laser-driven UPEB. Thus, considering the growing interest in the application of UPEBs in radiation biology and medicine, the aim of this study was to reveal the changes in the immune system in response to low-energy laser-driven UPEB whole-body irradiation in rodents. The adaptive response of the immune system of the organism to the ionizing radiation has a complex character. This is not only because of the complex network of organs, cells, and proteins that work together to provide an appropriate defensive response, but also because of the complexity of both their relationships and regulatory mechanisms. And therefore, in vitro studies alone are not able to provide sufficient insights into the adaptive response of the immune system of the organism and do not adequately reflect the processes taking part on the organismal level. This makes in vivo studies of the highest importance for the understanding of the radiation-induced alterations of the immune system of the organism.

## 2. Results

### 2.1. Peripheral Blood Profiling

**Figure 1:**
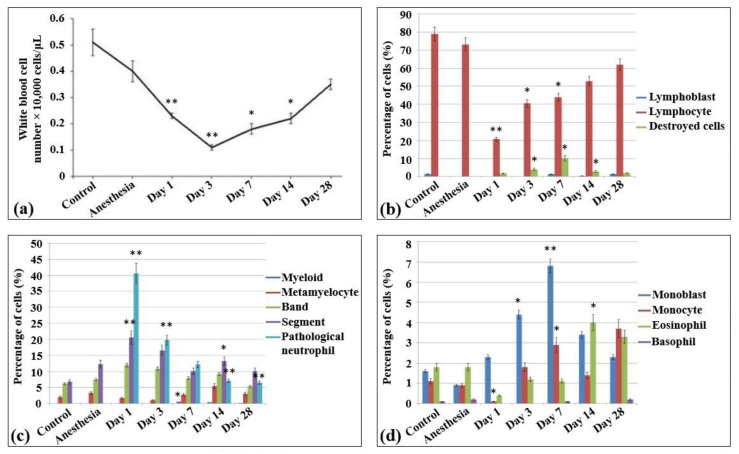
Cellularity and composition of white blood cells in the blood of control rats and after the irradiation with the laser-driven UPEB. (**a**) Total amount of white blood cells in peripheral blood of rats (µL). Percentages of (**b**) lymphoid and destroyed cells; (**c**) myelocytes and immature, mature, and pathological neutrophils; and (**d**) monoblasts, monocytes, eosinophils, and basophils. The number of rats in each group, n = 6. * *p* < 0.05; ** *p* < 0.001 (compared to control).

In the blood smears of control non-irradiated and non-anesthetized rats (Table S1), approximately 50 × 103 peripheral nuclear cells/µL were detected, all belonging to white blood cells (Figure 1a) and containing only monocytes, lymphocytes, and neutrophils (1–2%, 80%, and 15%, respectively). In the meantime, no nuclear non-mature erythroid forms, as well as no pathological and destroyed cells, were observed in these samples. The lymphoid population of these samples were almost represented by mature lymphocytes (Figure 1b). The lymphoblasts or prelymphocytes comprised only 1%. The blood myeloid population comprised 11–12% and mainly consisted of mature neutrophils (including an almost equal amount of banded (38.9%) and segmented (42) neutrophils), metamyelocytes (12.5%), and eosinophils (11.3%) (Figure 1c). An equal number of monocytes and monoblasts was detected (Figure 1d).

In general, the same peripheral blood profile was observed in the blood smears of the control non-irradiated rats subjected to anesthesia. Compared to control non-irradiated and non-anesthetized rats, the content of nuclear cells decreased slightly (41 × 103 peripheral nuclear cells/mL), while no difference was observed in the percentage content of the cells (monocytes accounted for 2%, lymphocytes for 75%, and neutrophils for over 23%).

The irradiation of animals with the laser-driven UPEB resulted in dramatic changes in the population content of blood after 24 h of irradiation. Thus, compared to both control groups (non-irradiated, non-anesthetized and non-irradiated, anesthetized animals), a statistically significant decrease was observed in the amount of white blood cells (17 × 103 cells/mL). Moreover, there was a 10–13 times statistically significant decrease in the percentage content of the lymphoid cells. Meanwhile, no changes were detected in the absolute number of neutrophils, while their relative abundance increased due to the loss of lymphoid cells (as a result, the content of neutrophils rose to 86.9% of all the peripheral nuclear cells). It should be mentioned that this type of irradiation also resulted in the appearance of a large population of pathological myeloid cells. Although there was a slight decrease in the absolute values of cell content in the population of monocytes, their relative abundance was increased 2 times (compared to control groups) due to the loss of the main amount of lymphocytes.

On the third day after irradiation, a termination in the progress of lymphopenia was observed, and the levels of lymphocytes did not differ from those observed on the first day after irradiation. Nevertheless, there was still a progress of leukopenia, which in this case was caused first of all by the loss of myeloid population resulting in the increase of the content of destroyed cells.

From the seventh day of irradiation, recovery processes of the cell populations were observed, including both absolute (the absolute content of blood cells was increased) and relative (the lymphoid population started to recover) recovery. This process continued up to the 14th and 28th day after irradiation, when almost all the indicators of white blood cells reached control levels. It should be noted that this distinctly expressed lymphopenia was not accompanied by the shift to the left, since the amounts of lymphoblasts in all the irradiation groups did not exceed those in the control group, i.e., no expressed regeneration processes took place.

**Figure 2:**
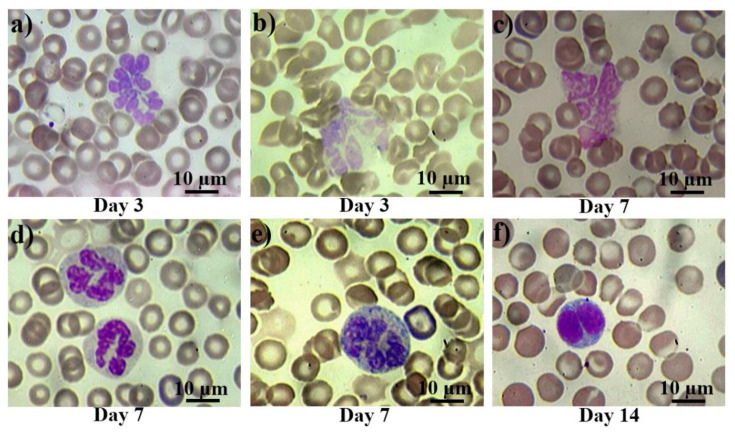
Pathological white blood cells in control rats and those exposed to the laser-driven UPEB irradiation. Hypersegmented (**a**) and pathological (**b**) giant neutrophils on the 3rd day after electron beam exposure; destroyed giant neutrophil (**c**), normally segmented giant neutrophils with basophilic cytoplasm (**d**), and severe basophilic granular cytoplasm (**e**) on the 7th day after irradiation exposure; and bilobed (binuclear) lymphocyte (**f**) on the 14th day after electron beam exposure. Blood slides were stained by Giemsa modified solution. Cells were examined under the light microscope at 1250× magnification. Scale bar is 10 μm.

The morphological changes of peripheral blood cells are presented in Figure 2. In control smears, there were either no pathological cells, or their number did not exceed 0.1% (they were observed mainly in the group of rats subjected to anesthesia). From the first day after the irradiation, atypical and pathological forms of leucocytes appeared in the blood of the rats. Then, bilobed (binuclear) lymphocytes occurred from the first to the third day (Figure 2a), reaching their peak on the seventh day after irradiation. Further, giant and hyper segmented neutrophils (Figure 2b,c) appeared on the third day, while destroyed cells (Figure 2d) appeared on the seventh day after irradiation. Also, diffuse basophilia in neutrophils occurred from the third to seventh day after irradiation exposure. Despite occasional severe cases (Figure 2f), in general, we can describe it as a moderate basophilia (Figure 2e).

### 2.2. Bone Marrow Profiling

**Figure 3:**
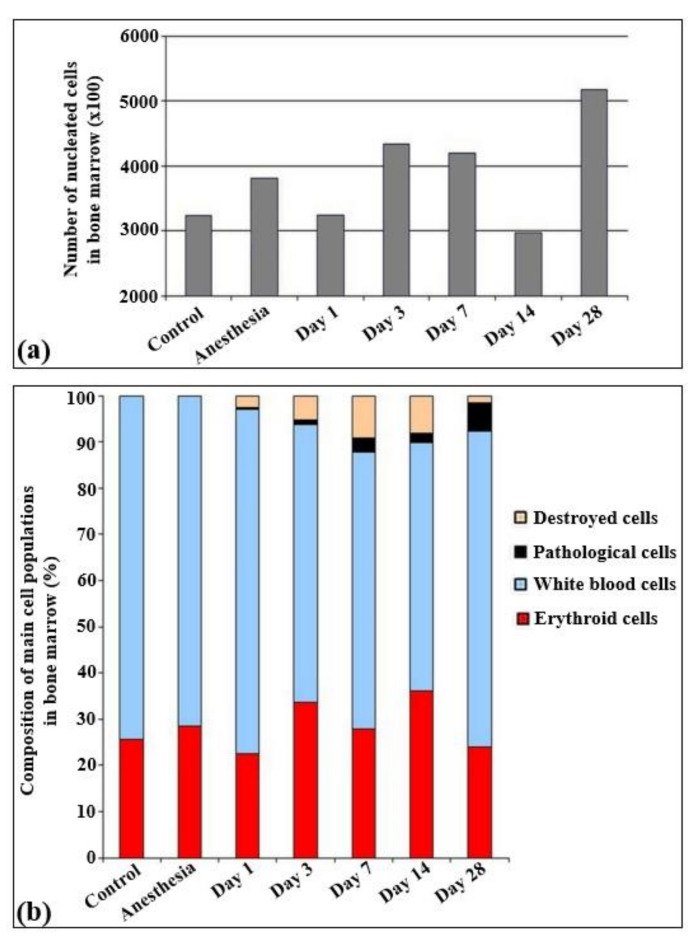
Cellularity and relative changes in the cell population of bone marrow in control rats and after the irradiation with the laser-driven UPEB. (**a**) Total amount of nucleated cells in bone marrow smears (×100). (**b**) Composition of main cell populations of bone marrow in control rats and after the influence of electron beam (%).

The total quantities of cells per unit of area are presented in Figure 3. According to the results obtained, statistically significant changes in the cell quantity were observed only on the 14th and 28th day of irradiation, Particularly, the cell quantity statistically decreased on the 14th day and increased on the 28th day after irradiation.

**Figure 4:**
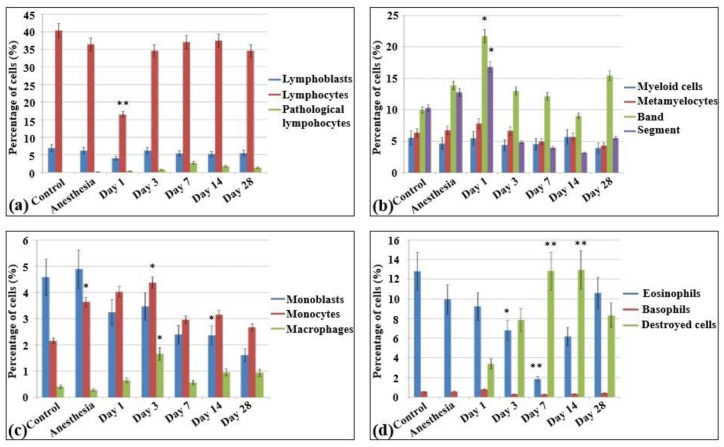
Cellularity and composition of white blood cells in bone marrow smears in control rats and after the irradiation with the laser-driven UPEB. Percentages of (**a**) lymphoid cells; (**b**) myelocytes, and immature and mature neutrophils; (**c**) monoblasts, monocytes, and macrophages; and (**d**) eosinophils, basophils, and destroyed cells. * *p* < 0.05; ** *p* < 0.001 (compared to control).

The immune cells were represented by lymphoid (30–35%), myeloid (32–35%), and monocytic (5%) populations. All other cells overall constituted less than 1%. The analysis of the myelogram in both control groups of rats (non-irradiated, non-anesthetized and non-irradiated, anesthetized animals) revealed that in these groups the content of red blood elements was about 25–30% of all the parenchymal cells (Figure 4).

**Figure 5:**
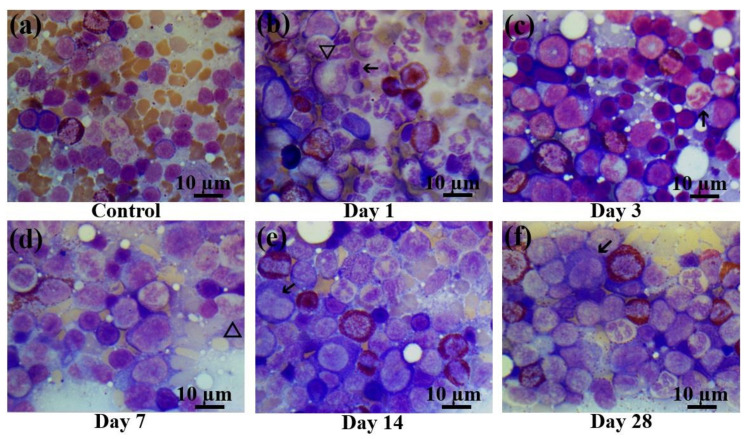
Bone marrow smears in control rats and those exposed to the laser-driven UPEB irradiation. (**a**) Control bone marrow smears. (**b**) 1st day after electron beam exposure: prominent proliferation of myeloid cells; lymphopenia, occurrence of destroyed cells. Pathological cells (arrowed) and giant neutrophils (triangle). (**c**) 3rd day after electron beam exposure: increased number of erythroid cells, lack of mature leucocytes; pathological neutrophil (arrowed). (**d**) 7th day after electron beam exposure: partial recovery of leucocyte population. Presence of giant neutrophils, with basophilic cytoplasm (triangle). (**e**) 14th day after electron beam exposure: partial recovery of lymphocyte population. Presence of pathological bilobed lymphoblasts (arrowed). (**f**) 28th day after electron beam exposure: recovery of leucocyte population; presence of pathological lymphocytes (arrowed). Bone marrow smears were stained by Giemsa modified solution. Cells were examined under the light microscope at 1250× magnification. Scale bar is 10 μm.

In the bone marrow cellular populations, a significant decrease in the content of mature lymphocytes was observed on the first day after irradiation, with a simultaneous increase in the content of pathological lymphocytes and the appearance of a large number of destroyed cells. Meanwhile, there was a statistically significant increase in the content of late myeloid cells, from metamyelocytes to segmented neutrophils (Figure 5b). Then, a statistically significant decrease of the content of mature neutrophils was observed on the third (Figure 5c) and seventh day (Figure 5d) with no significant changes in the amount of more early forms. The percentage content of the lymphoid cells reached the same level as that of the control group starting on the third day. Here, a decrease in the content of monoblasts was observed for the first time, and pathological erythroblasts occurred since the third day. The proliferation of erythroid cells became visible on the third day after electron beam exposure (Figure 5c). In contrast to the blood smears, the cellular populations in the bone marrow recovered only partially from the 14th (Figure 5e) to the 28th day (Figure 5f). Although the cell populations in the bone marrow reached the control levels, nevertheless, pathological cells were observed in all populations (lymphoid, myeloid, and erythroid), sometimes even in quite substantial numbers. Meanwhile, nearly 6% of the bone marrow cells displayed signs of apoptosis. The populations of monoblasts and segmented neutrophils did not reach the control levels even on day 28 (Table S2).

### 2.3. Lymph Node Profiling

**Figure 6:**
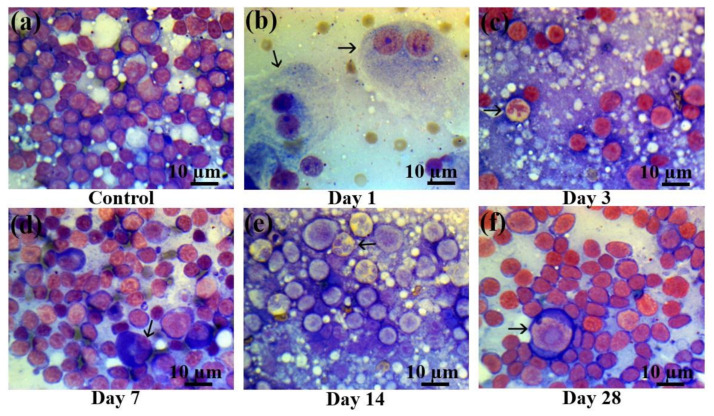
Lymph node smears in control rats and those exposed to the laser-driven UPEB irradiation. (**a**) Control lymph node smears; (**b**) 1st day after electron beam exposure: prominent lymphopenia arising from destroyed cells; pathological binuclear macrophages (arrowed); (**c**) 3rd day after electron beam exposure: increased amount of neutrophils (arrowed); lymphopenia; (**d**) 7th day after electron beam exposure: partial recovery of lymphocyte population; pathological neutrophil (arrowed); (**e**) 14th day after electron beam exposure: partial recovery of lymphocyte population; presence of pathological bilobed lymphoblasts (arrowed). (**f**) 28th day after electron beam exposure: partial recovery of lymphoid population; presence of proerythroblasts (arrowed). Lymph node smears were stained by Giemsa modified solution. Cells were examined under the light microscope at 1250× magnification. Scale bar is 10 μm.

As in the bone marrow, a statistically significant decrease in the content of mature lymphocytes was observed in the lymph nodes (Figure 6) on the first day of electron beam irradiation, compared with the control group (Figure 6a), which was accompanied by an increase in the content of pathological lymphocytes and the appearance of a large number of destroyed and pathological cells (Figure 6b). Severe lymphopenia and an increased number of myeloid cells were observed on the third day after electron beam exposure (Figure 6c). Then, partial recovery of the lymphoid population took place on day 7 after electron beam exposure (Figure 6d), which continued up to day 14 (Figure 6e). On the 28th day after electron beam exposure, there was a reduction of the content of myeloid cells (Figure 6f) and an almost full recovery of the lymphoid population with the occasional presence of early blast cells (Table S3).

### 2.4. Production of Proinflammatory and Anti-Inflammatory Cytokines in Response to the Laser-Driven UPEB Irradiation

**Figure 7:**
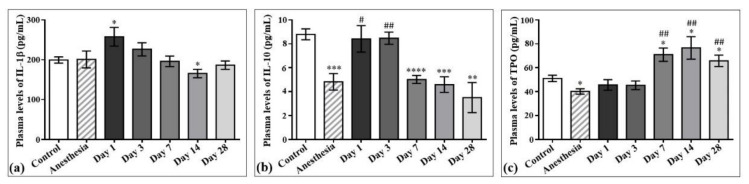
The effect of laser-driven UPEB exposure on IL-1β, IL-10, and thrombopoietin (TPO) levels in the plasma samples of control rats and those exposed to irradiation. Plasma levels of IL-1β (**a**), IL-10 (**b**) and TPO (**c**) in control and irradiated rats measured on the 1st, 3rd, 7th, 14th, and 28th day after irradiation. * *p* < 0.05; ** *p* < 0.01; *** *p* < 0.001; **** *p* < 0.0001; # *p* < 0.05; ## *p* < 0.01 (* compared to control; # compared to anesthesia).

The concentrations of interleukin-1β (IL-1β) and interleukin-10 (IL-10) in the plasma samples of the control rats and those exposed to the laser-driven UPEB irradiation (Figure 7) were also measured. According to the results obtained, the plasma levels of IL-1β significantly increased on day 1 (Figure 7a) and decreased on day 14 after irradiation. Regarding IL-10, its levels significantly decreased from the 7th to the 28th day after electron beam irradiation compared to the control group, and did not return to control levels even on the 28th day after electron beam exposure (Figure 7b).

### 2.5. The Effect of Laser-Driven UPEB Irradiation on DNA Damage and Repair in Selected Organs of the Hematopoietic System

**Figure 8:**
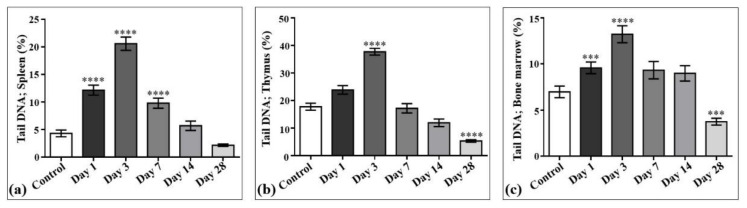
DNA damage induced by low-energy ultrashort-pulsed electron beam irradiation in vivo in rats; results of comet assay in spleen (**a**), thymus (**b**), and bone marrow (**c**) cells. *** *p* < 0.001; **** *p* < 0.0001 (compared to control).

**Figure 9:**
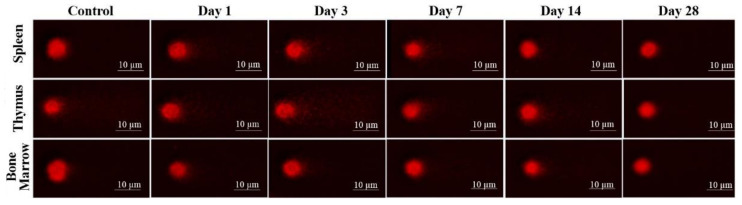
DNA fragmentation detected in spleen, thymus, and bone marrow (BM) cells by comet assay after low energy ultrashort-pulsed electron beam irradiation *in vivo* of rats. Scale bar is 10 μm.

The results of laser-driven UPEB irradiation-induced DNA damage in the cells obtained from spleen, thymus, and bone marrow are presented in Figure 8, and the representative images of the comet assay are shown in Figure 9. In the irradiated animals, the level of DNA damages in the spleen (Figure 8a and Figure 9), thymus (Figure 8b and Figure 9), and bone marrow (Figure 8c and Figure 9) cells increased up to the third day of electron beam exposure. The spleen was shown to be the most sensitive organ, since the level of DNA damages increased by around five times when compared to the control group. The thymus and bone marrow cells exhibited almost equal sensitivity compared to that of the corresponding controls (a two-fold increase).

The viability of cells, assessed in parallel to each experimental point, was not significantly changed compared to that of the corresponding controls, being 75.8 ± 3.21% in the spleen, 89.2 ± 1.56% in the thymus, and 86.3 ± 2.78% in the bone marrow cell populations. A visible but not statistically significant decrease in the weight of the thymus and spleen was observed. Particularly, the mean absolute weight decrease of approximately 16% in the thymus was detected on the 3rd day (435 ± 40.8 mg) and by 9.9% on the 28th day (472 ± 24.53 mg) of the study, compared to that in the control group (524 ± 68.12 mg). The mean absolute weight of the spleen was slightly decreased (5.9%) on the seventh day (785 ± 56.5 mg) of the study, as compared with the control value (834 ± 93.4 mg). The DNA damage recovery was most effective in the thymus, then in the bone marrow, and to the least extent in the spleen cells. However, on the 28th day of the study, the DNA damage levels reached the control (spleen) or even lower (thymus and bone marrow) values.

## 3. Discussion

In this manuscript, we studied the laser-driven UPEB whole-body irradiation-induced radiation adaptive response in the immune system of *Wistar* rats. We tested the effect of laser-driven UPEB whole-body irradiation on the cells of peripheral blood, bone marrow, and lymph nodes, as well as cytokine levels in peripheral blood plasma and DNA damage and repair in the spleen, thymus, and bone marrow of rats. The number of animals involved in each of the seven experimental groups of this study allowed us to obtain statistically reliable and significant data on the dynamics of the alterations of the studied parameters of the immune system during 28 days after radiation exposure, to assess the radiosensitivity of different organs and blood and bone marrow cells to the laser-driven UPEB whole-body irradiation. The data obtained describes for the first time the adaptive reactions of the immune system of rats in response to the irradiation with laser-driven UPEBs. This study could be used as a basis for future, more detailed investigations of the molecular and cellular mechanisms of these reactions, as well as for the assessment of the effect/efficiency of different radioprotective agents on the immune system of the animals.

In general, whole-body irradiation studies are important for understanding the extent to which the radiation causes different effects in the organism, whether it is whole-body irradiation-induced injury from a radiation accident or a radiological terrorism event (including atomic bombings or serious nuclear power plant accidents), or if it is a conditioning therapy before hematopoietic stem cell or bone marrow transplantation for the treatment of several types of cancer, including leukemia, lymphoma, and multiple myeloma [26,27]. Thus, whole-body irradiation studies can be used as an evidence-based knowledge in the field of medicinal sciences (in the radiobiological studies of the potential efficiency of the application of this new type of irradiation in clinical practice) and in environmental sciences and environmental bioengineering. For example, whole-body irradiation is often used in cancer therapy in combination with high-dose anticancer drugs to help prepare a patient for a stem cell transplant. In practice, irradiation of the whole body is typically fractionated, with smaller doses delivered in several sessions, rather than delivering the entire dose at once. Early research in bone marrow transplantation demonstrated that the application of multiple small doses resulted in lower toxicity and better outcomes than delivering a single, large dose [27].

We revealed statistically significant immune cell depletion and recovery consequent to total body irradiation by laser-driven UPEB. First of all, we studied the changes in the main indicators of the immune system after the laser-driven UPEB irradiation. Such studies are limited technically due to the importance of laser-driven UPEBs produced by linear accelerators. In a linear accelerator, electrons are accelerated to a high energy and can be used in some medical areas. There are several properties which make the laser-driven UPEBs promising for future therapeutic approaches. First of all, this new kind of electron beam irradiation is very promising in different fields of clinical oncology, not only because of the smaller penetration rate of electron beams into deeper situated tissues, but also because laser-driven electron linear accelerators provide an opportunity to regulate the penetration rate by modifying the energy of the incident electrons [28].

The studies of the immune cell pathology via investigation of blood cell populations revealed a statistically significant pancytopenia in peripheral blood with relative neutrophilic leukocytosis with a slight shift of the leukocyte formula to the left, indicating absolute and relative lymphopenia. The reason for the progress of leukopenia, caused by the loss of myeloid population and resulting in the increase of the content of destroyed cells, may be apoptosis. These results are in compliance with the first stage of acute radiation syndrome (sickness) to the primary acute reaction on the acute radiation injury. In general, there are four phases of acute radiation injury (prodromal, latent period, manifest illness stage, and recovery or death) based on which the course of the syndrome varies from several hours to several weeks depending on the irradiation type (X-ray, electron beam, etc.), the dose, the part of the body exposed to irradiation (e.g., whole-body irradiation, partial-body irradiation, organ irradiation, etc.), and different individual characteristics (age, gender, medical conditions, etc.) [29].

Young and rapidly growing tissues are the most radiosensitive ones to radiation exposure [30]. Representing the first line of such tissues, bone marrow is known as the most radiation-sensitive organ in mammalian hematopoietic systems. Thus, it has been shown that X-ray irradiation results in the lack of almost all cells in bone marrow with the presence of only connective tissue and fat cells, blood vessel endothelium, phagocytes, and occasional normoblasts [31]. According to another study, such a depletion of bone marrow cells and hematopoietic progenitor cell reservoirs rapidly and dramatically decreases the number of circulating blood cells [32,33]. Our investigations showed statistically significant decreased amounts of all types of white blood cells and increased levels of destroyed and pathological cells since the first day after laser-driven UPEB exposure. At early stages after exposure, main changes occur as result of severe lymphopenia. Lymphopenia is a leading symptom after ionizing radiation [34]. The statistically significant decrease in cell number on the 14th day may have been due to cell transfer from bone marrow aiming to recover the blood cell population (Figure 1), and the increase on the 28th day may have been due to the increased bone marrow regeneration.

The lymph node is a dynamic organ, composed of transient lymphocytes, a small number of monocytes and/or macrophages, several lymphoblasts, and very rarely erythroblasts. After electron beam exposure, a severe lymphopenia and a significant number of destroyed and pathological cells occurred. Partial recovery occurred on the 7th day after electron beam exposure, which continued up to the 14th and the 28th day.

According to the obtained results, it can be concluded that after the whole-body irradiation by the laser-driven UPEBs, a partial recovery of the immune system took place in a shorter time, unlike with X-ray or gamma ray radiation [35,36,37,38]. Usually, the recovery of the content of lymphocytes in the blood is quite a slow process: it reaches the control levels only several months after irradiation. The statistically significant decline in the levels of lymphocytes takes place in part due to the alteration in the reproductive activity of the precursor cells, but mostly due to the direct lympholytic effect of the ionizing radiation.

The formation of bilobed lymphocytes after electron beam exposure is an interesting phenomenon, which was first described by cyclotron personnel at the University of Rochester in 1948–1951 [39], and then in humans, who received different doses of radiation [34,35,36,37,38,39,40,41,42,43].

As described previously [44], the formation of giant neutrophils reflects the proliferative response of the bone marrow. It was shown that in acute radiation syndrome the proliferative activity and differentiation rate of myeloid cells decrease at early stages after irradiation [45]. Moreover, giant neutrophils are formed from myeloblasts, being at the most active phase of the S stage at the time of irradiation and having a very low differentiation rate. In all cases, these cells represent the most common expression of dysgranulopoiesis [46]. Moreover, after irradiation, neutrophils demonstrate basophilic staining of cytoplasm. As known, basophilic staining of cytoplasm is the manifestation of an increased amount of ribosomal RNA. Diffuse basophilia in neutrophils occurs when ribosomes continue to stay scattered throughout the cytoplasm instead of being degraded during maturation. This is usually described as the pathology of maturation or toxic effects [47,48].

The delayed immune reconstitution is known as a major cause of morbidity. It has been shown to be associated with myelosuppression, which is one of the detrimental effects of cytotoxic radiation therapy. Therefore, the reconstitution of all immune cells using hematopoietic stem cells, as well as T-cell recovery via the effective thymopoiesis, could be an efficient therapeutic strategy for the restoration of the functional immune cell repertoire [49].

It is well-known that ionizing radiation leads to the alterations in the expression of proinflammatory and anti-inflammatory cytokines, which is highly time-dependent and occurs as a response of the organism to the radiation in the form of cytokine storming with a subsequent total or partial recovery to baseline levels [50,51], depending on the murine strain and radiation type [52,53]. Such a storming phenomenon typically occurs after many stress situations and seems to have a regulating role on immune responses, as well as inflammatory and growth signaling cascades aimed at improving host response [53]. According to our data, we registered increased plasma levels of IL-1β, which occurred on the first day after laser-driven UPEB exposure, preceding the increase in lymphocyte levels after lymphopenia, and probably stimulating this growth. The increase in IL-1β levels was accompanied by the simultaneous severe lymphopenia on the first day after laser-driven UPEB exposure, and the decreased levels occurred after the beginning of the recovery of the lymphoid population. The IL-1 family of cytokines has been shown to play key roles in inflammatory and immune responses [54], which allows these cytokines to be considered as the first line of host defense against stresses [52,53,54].

Decreased amounts of IL-10 from the seventh day after laser-driven UPEB exposure revealed in this study can explain the phenomenon of the removal of mature neutrophils (bands and segments) from the bone marrow since the seventh day, as well as the absence of the rise of these cells in the blood on the same day. It is known that anti-inflammatory interleukins, such as IL-10, inhibit the expression of endothelial adhesion molecules and prevent the leukocyte extravasation, thereby preventing leukopenia [55].

The comet assay applied in this study is widely used in different biomedical fields, including radiation biology, toxicology, and oncology. It is a single-cell gel electrophoresis tool for the determination of DNA damage and repair kinetics [56,57]. X-ray irradiation-induced DNA damage has been reported in different organisms, organs, and cell types using this approach [58,59,60]. In this study, we explored the extent of DNA damage in the spleen, thymus, and bone marrow of *Wistar* rats induced by whole-body laser-driven UPEB irradiation. The hematopoietic system, including the bone marrow, spleen, thymus, and lymph nodes, is highly sensitive to ionizing radiation. Since DNA is the primary target of ionizing radiation, we performed comet assay on the cells obtained from the spleen, thymus, and bone marrow, to compare the tissue sensitivity and possible adverse effect of low-energy UPEB irradiation. While the spleen was the most sensitive to low-energy UPEB radiation, demonstrating a higher level of DNA damages and slower recovery kinetics, a drastic increase in DNA damages in the thymus and bone marrow cells was also observed. It is well known that hematopoietic organs are mutually related, which is expressed by the consequent cell migration from the bone marrow to the thymus and then to the spleen and lymph nodes. Thus, the slight decrease in the mean absolute weight of the spleen with the persistent high level of DNA damages, detected on the 7th day, can be explained by the death of highly damaged cells, whereas the delayed recovery of the spleen, observed on the 14th day of the study, can be associated with the recovery kinetics of bone marrow and thymus (day 7–14), the time period until irradiated bone marrow can seed the spleen with the sufficient number of non-damaged cells. The biphasic pattern of thymus weight change (on the 3rd and 28th day) can be explained by the death of highly damaged cells during the first three days after irradiation, with the consequent proliferation of surviving cells in the thymus. However, the second slight decrease of spleen weight, while the cells’ DNA was shown to be undamaged, can be associated with the insufficient supply of cells from the bone marrow, which itself demonstrated a significant decrease of DNA damages in cells during day 14–28 of the study. The non-detected changes in cell viability in the parallel experiments could be due to the loss of dead cells during cell isolation procedures (e.g., centrifugation) or apoptosis. Overall, DNA damages, observed in the bone marrow, thymus, and spleen, achieved the maximal level on the 3rd day of the study, with further recovery on the 7th day (in the case of the bone marrow and thymus) and on the 14th day (in the case of the spleen) after irradiation. The lower levels of DNA damages compared to those of the corresponding controls, observed in the spleen and bone marrow cells, on the 28th day after irradiation, can be explained by the full repopulation of those organs with healthy cells.

The interplay between ionizing radiation-induced DNA damages and immune system alterations has been shown earlier, which was explained by the activation of DNA damage response machinery leading to the immunogenicity of the damaged cells. The reciprocal/reverse situation was also observed. For instance, the prolonged expression of cytokines, such as interferon β (IFNβ) or interferon γ (IFNγ), led to the accumulation of DNA double-strand breaks [61]. The role of the immune system in the propagation of genotoxic effects was shown for localized irradiation with high-dose-rate synchrotron low-linear energy transfer radiation, which was attributed to the non-targeted radiation-induced abscopal effects [62]. Our results support the idea of close communication between irradiation-induced DNA damages observed in hematopoietic system organs/tissues and components of the immune system, although the causal relationship and molecular mechanisms in the case of UPEB irradiation still need to be explored during further localized radiation exposure experiments on tumor-bearing animals.

With regard to the development perspective, experiments have to be done to understand the effect of this novel type of irradiation on the biological level, pathological and molecular alterations, and different systems and organs of the animal, including the oxidant/antioxidant system, lipid metabolism, cell death mechanisms, DNA alterations, and so on.

## 4. Materials and Methods

### 4.1. Animals

Forty-two male albino *Wistar* rats weighing approximately 180–200 g were involved in this study. The rats were housed and cared for under controlled colony conditions providing 12-h reverse light–dark cycles in a temperature-regulated animal facility (22 °C). Rats had access to food and water ad libitum. All the rats were fed a standard laboratory chow diet (B&K Universal). The rats were randomly divided into seven groups: a control group of six non-irradiated animals (necessary for the assessment of the initial native levels of the studied parameters in the non-irradiated rats), a sham group of six non-irradiated anesthetized animals (necessary for the assessment of the effect of the anesthesia only on the studied parameters in the non-irradiated rats), and five irradiation groups each consisting of six animals (necessary for the assessment of the dynamics of the alterations of the studied parameters during the 28 days after radiation exposure). The number of animals was chosen and designed as *n* = 6 to correspond to the minimum amount of the animals necessary to conduct an adequate and well-controlled study to perform the accurate and reliable statistical analysis of the data obtained.

The animal protocol was approved by the Animal Ethical Committee of the Institute of Molecular Biology NAS RA and the experiments were carried out in accordance with its relevant guidelines and regulations. All the rats except those in the control group were anesthetized with an intramuscular administration of 20 mg/kg aminazine (chlorpromazine hydrochloride, Sigma-Aldrich, Taufkirchen, Germany). The adequate depth of the anesthesia was ensured by testing the pedal withdrawal and palpebral reflexes. All the rats were euthanized by carbon dioxide overdose followed by blood collection from the carotid artery and subsequent decapitation in the morning of the 1st, 3rd, 7th, 14th, and 28th day after irradiation and on the 1st day after anesthesia in the sham group.

### 4.2. Irradiation Source

As an irradiation source, the laser-driven UPEB from AREAL (Advanced Research Electron Accelerator Laboratory) facility was used developed at CANDLE (Center for the Advancement of Natural Discoveries using Light Emission) Synchrotron Research Institute, Yerevan, Armenia. Radiation treatment was carried out using an electron beam generated by a laser-driven radiofrequency gun-based linear AREAL accelerator. To extract the electron beam from the accelerator, a 20 μm thick titanium window was mounted at the end of the experimental beamline. The characteristics of the AREAL accelerator, as well as the description of the dose calibration measurements and uniformity quality assurance, have previously been described [22,24,47,63]. The facility provides stable operation using an electron beam with well-formed and reproducible characterizing parameters. All machine parameters were measured and compared to previous ones before every experiment. The parameters of the AREAL laser-generated electron beam are presented in Table 1.

### 4.3. Whole Body Irradiation by Laser-Driven UPEB

The one-time irradiation was delivered at 10:00–11:00 AM with a dose of 2 Gy and a repetition rate of 2 Hz corresponding to the lethal dose 50 (LD50) for this type of irradiation and animal, determined previously [63]. The exposure time was calculated for each rat according to their weight, which can be estimated as approximately 9–10 min for 180–200 g of the animal. The rats were positioned on a homemade wooden frame to avoid any electrical phenomena, held vertically in the radiation path, and anterior total body irradiation was delivered. The beam field size was set to a 40 cm diameter with a source-to-skin distance (SSD) of 40 cm.

### 4.4. Experimental Design

The rats were divided into seven groups with six rats in each group. There were five irradiated groups exposed to laser-driven UPEB irradiation and euthanized on the 1st, 3rd, 7th, 14th, and 28th day after irradiation, as well as the control non-irradiated group. Peripheral blood, bone marrow, and lymph node profiling was conducted. The levels of proinflammatory and anti-inflammatory cytokines were determined in peripheral blood plasma samples, and DNA damage and repair was assessed in the spleen, thymus, and bone marrow of the rats.

### 4.5. Sample Collection

#### 4.5.1. Blood Sample Collection

Blood samples (4 mL) were collected from the tail vein of the rate into ethylenediaminetetraacetic acid-containing (EDTA-containing) tubes (to prevent coagulation) for the isolation of plasma samples and a complete blood picture and blood film morphology. For the isolation of plasma samples, the blood samples were kept on ice for 60 min with the following centrifugation at 3000× *g* for 15 min at 4 °C; they were then put in storage at −30 °C until further use. Immediately prior to use, the plasma samples were thawed and centrifuged at 10,000 rpm for 5 min at 4 °C to remove any precipitate. For complete blood cell count analysis, conventional blood smears were made by spreading a blood drop on a clean slide with another one with subsequent air drying and Giemsa staining. The films were viewed under the light microscope using the 100× objective for counting.

#### 4.5.2. Bone Marrow Imprints

Bone marrow was obtained from the humerus, as described previously [64], and the absolute number of nucleated cells was counted with the standard cell counter. Morphological analysis of the bone marrow cells was performed on smears obtained from the contralateral humerus, and differential counts were performed according to the standard morphologic criteria for rats, as reported previously [65]. For cell analysis, slides were fixed in pure methanol and stained with modified Giemsa solution (azure B/azure II, eosin, and methylene blue) according to the manufacturer’s protocol (Sigma-Aldrich, Taufkirchen, Germany). Cells were analyzed and counted in 100 randomly selected fields (0.01 mm^2^) using a light microscope at 1250× magnification. In total, at least 3000 cells were analyzed and classified at a given day before and after radiation.

#### 4.5.3. Isolation of Bone Marrow Cells

Bone marrow cells were isolated according to the previously described procedure [66]. Briefly, rat femurs were placed in a petri dish containing 5 mL of cold sterile bone marrow medium (500 mL of Iscove’s Modified Dulbecco’s Medium (IMDM) with 2% fetal bovine serum (FBS), 2 mM of L-glutamine, and 100 mg/mL of penicillin/streptomycin) and trimmed to expose the interior marrow shaft of the femur; the end of the femur was then cut off. A cc syringe with a 25-gauge needle was used to flush the bone marrow medium through the femur several times to release cells into the petri dish. The suspension was immediately transferred to a 15 mL centrifuge tube, centrifuged at 200× *g* for 10 min, aspirated, and washed with bone marrow medium. The cell count and viability were determined by Trypan blue exclusion tests [67].

#### 4.5.4. Lymph Node Imprints

Fresh lymph nodes (from the abdominal cavity) were cut with a sharp blade through the hilum, and tissue imprints were made by gently touching the freshly cut surface of the tissue with a clean glass microscope slide. For cell analysis, slides were fixed in pure methanol and stained with modified Giemsa solution (azure B/azure II, eosin, and methylene blue) according to the manufacturer’s protocol (Sigma-Aldrich, Taufkirchen, Germany). Cells were analyzed and counted in 100 randomly selected fields (0.01 mm^2^) using a light microscope at 1250× magnification. In total, at least 3000 cells were analyzed and classified at a given day before and after radiation.

#### 4.5.5. Isolation of Thymus and Spleen Cells

The thymuses and spleens were isolated and weighed following the procedures described previously [68]. Briefly, the rat thymuses and spleens were harvested in an animal facility and transferred to the laboratory in HBSS on ice. Single cell suspensions of spleen and thymus cells were prepared by homogenizing the organ between the frosted ends of two sterilized microscope slides (Fisher Scientific, Pittsburgh, PA, USA) into a dish containing 5 mL of cold sterile rat medium (500 mL RPMI 1640 (Roswell Park Memorial Institute medium) with 10% FBS, 2 mM of L-glutamine, and 100 mg/mL of penicillin/streptomycin). Suspended cells were centrifuged at 200× *g* for 10 min, aspirated, and washed with rat medium. The cell count and viability were determined by Trypan blue exclusion tests [67].

### 4.6. Determination of IL-1β, IL-10, and Thrombopoietin Levels in Plasma

The levels of the proinflammatory and anti-inflammatory cytokines IL-1β, IL-10, and thrombopoietin were determined using commercially available ELISA (enzyme linked immunosorbent analysis) kits (Bioassay Technology Laboratory, Shanghai, China) according to the manufacturers’ instructions. The concentrations of IL-1β and IL-10 were expressed in pg per mL of plasma (pg/mL), and the concentrations of thrombopoietin were expressed in ng per mL of plasma (ng/mL).

### 4.7. Comet Assay

The alkaline comet assay was performed according to the previously described method [69]. Bone marrow, spleen, and thymus cells at the concentration of 1 × 10^6^ cells/mL were mixed with low-melting-point agarose (0.5%) (20:180 μL) at 37 °C, placed on slides pre-coated with a layer of normal melting agarose (0.5%), and allowed to polymerize at 4 °C for 5 min. After removing the cover slips, the slides were placed in Coplin jars with 50 mL of lysis solution (1% Triton X-100, 10% dimethyl sulfoxide, and 89% 2.5 M NaCl, 100 mM EDTA-Na2, and 10 mM Tris, pH > 10) at 4 °C. After 1 h of incubation in lysis solution, the slides were placed in a horizontal gel electrophoresis tank side by side, leaving no spaces. The slides were incubated in a fresh alkaline (pH 13) electrophoresis buffer (300 mmol/L of NaOH and 1 mmol/L of EDTA-Na2) for 20 min to allow for the unwinding of DNA, and then electrophoresis was run at 25 V and 300 mA for 20 min at 4 °C. Slides were neutralized three times for 5 min in 0.4 mol/L of Tris, pH 7.5, and DNA was stained with 20 μL of 20 μg/mL ethidium bromide. Cells were analyzed using a Nikon Labophot 2 fluorescence microscope (Nikon, Tokyo, Japan) at 200 × magnification and computer-aided image analysis. Images of at least 100 cells (50 cells from each of the two slides) were evaluated by the use of the software program Komet 5 (BFI Optilas, Gröbenzell, Germany). The percentage of DNA in the tail region of each cell was determined (tail DNA, %).

### 4.8. Statistical Analysis

Statistical analysis was performed using GraphPad Prism 5.01 (GraphPad Software, San Diego, CA, USA). Depending on parametric or non-parametric data, one-way ANOVA or the Kruskal–Wallis tests were used, and the differences between groups were determined with Bonferroni or Dunn’s post-hoc tests, respectively. Values are expressed as mean ± standard error of the mean (SEM), and *p* < 0.05 was considered as the statistically significant value.

## 5. Conclusions

In conclusion, the studies of the biological effect of laser-driven UPEB whole-body irradiation showed that this type of irradiation induces alterations in the rat immune system, particularly by increasing the production of pro- and anti-inflammatory cytokines and elevating the DNA damage rate. Moreover, such an immune response reaches its maximal levels on the third day after laser-driven UPEB whole-body irradiation, showing partial recovery on subsequent days with the total recovery on the 28th day. The adaptive reactions of the immune system of rats are described for the first time in response to the irradiation with laser-driven UPEBs. This study could be used as a basis for future, more detailed investigations of the molecular and cellular mechanisms of these reactions, as well as for the assessment of the effect/efficiency of different radioprotective agents on the immune systems of animals. The results of this study provide valuable insight into the effect of laser-driven UPEB whole-body irradiation on the immune systems of animals and supports further animal experiments on the role of this novel type of irradiation. This understanding will serve as a background and will be necessary for future studies of possible applications of laser-driven UPEB irradiation in cancer treatment strategies as a promising new radiotherapeutic approach, mitigating the risk of the harmful side effects of radiation therapy and providing early recovery of the organism afterwards, as well as for ensuring that the adoption of this promising technology is appropriate and evidence based.

## Figures and Tables

**Table 1:** Characteristics of the AREAL laser-generated electron beam.

AREAL Beam Parameters		UV Laser Parameters	
Beam charge @ experiment (pC)	30	Wavelength (nm)	258
Electron energy (MeV)	3.6	Pulse energy (μJ)	~500
Pulse duration (fs)	450	Repetition rate (Hz)	2
Pulse repetition rate (Hz)	2	Energy stability (%)	<0.1
Beam spot (mm)	15	Beam divergence (mrad)	<0.1
Norm. transv. emittance (mm-mrad)	<0.5	Spot size @ cath. (mm)	2.0
RMS (root-mean-square) energy spread (%)	<1.5		
Online dose information	Farady cup/dosimeter		

## Data Availability

Not applicable.

## Supplementary Materials

The following are available online at https://www.mdpi.com/article/10.3390/ijms222111525/s1.
