# Supplementary material for: Low-Energy Laser-Driven Ultrashort Pulsed Electron Beam Irradiation-Induced Immune Response in Rats
Source: Int J Mol Sci. 2021 Oct 26;22(21):11525. doi: 10.3390/ijms222111525 (PMC8584044; doi:10.3390/ijms222111525)
Supplement: Supplementary file 1 [file ijms-22-11525-s001.zip › ijms-1375657-supplementary.pdf]

Table S1. blood immune cell populations in control rats and those exposed to the laser driven UPEB irradiation.

Cells	Control (M ± SD)	Anesthesia (M ± SD)	1st day (M ± SD)	3rd day (M ± SD)	7th day (M ± SD)	14th day (M ± SD)	28th day (M ± SD)
Lymphoblast	1.4 ± 0.3	-	-	-	1.3 ± 0.3	0.3 ± 0.1*	1.3 ± 0.4
Lymphocyte	79 ± 4.9	73.1 ± 6.1	20.7 ± 1.8*	40.5 ± 5.3*	43.9 ± 4.7*	52.8 ± 7.7*	61.9 ± 8.1*
Monoblast	1.6 ± 0.2	0.9 ± 0.1	2.3 ± 1.0	4.4 ± 0.8**	6.8 ± 1.2**	3.4 ± 0.7**	2.3 ± 0.5
Monocyte	1.1 ± 0.2	0.9 ± 0.1	-	1.8 ± 0.3	2.9 ± 0.2	1.4 ± 0.1	3.7 ± 0.3
Mieloid	-	-	-	-	0.6 ± 0.1	0.4 ± 0.1	0.2 ± 0.1
Metamielocyte	2.0 ± 0.4	3.3 ± 0.5	1.7 ± 0.1	1 ± 0.1*	2.8 ± 0.3	5.5 ± 0.8**	3.1 ± 0.8**
Band	6.2 ± 0.6	7.5 ± 0.9	12 ± 0.8**	10.9 ± 1.0**	7.9 ± 1.1	9.2 ± 1.2**	5.4 ± 0.9
Segment	6.8 ± 0.5	12.3 ± 0.9	20.6 ± 2.1**	16.6 ± 2.2**	10.1 ± 1.2**	13.3 ± 2.0**	10.1 ± 0.9**
Pathol neutrophil	-	-	40.6 ± 8.3**	19.8 ± 5.4**	12.2 ± 2.4**	7.1 ± 2.3**	6.5 ± 0.7**
Eosinophil	1.8 ± 0.2	1.8 ± 0.4	0.4 ± 0.1*	1.2 ± 0.2	1.1 ± 0.2	4.0 ± 1.3	3.3 ± 0.5
Basophil	0.1 ± 0.03	0.1 ± 0.1	0.1 ± 0.1	0.01 ± 0.01	0.2 ± 0.01	0.01 ± 0.01	0.2 ± 0.01
Destroyed	-	-	1.7 ± 0.5**	3.9 ± 0.6**	10.1 ± 2.4**	2.7 ± 0.8**	1.9 ± 0.4**

Legend: *significantly lower compared with controls or tendency ($p < 0.1 – p < 0.01$); **significantly higher compared with controls or tendency ($p < 0.1 – p < 0.01$).

Table S2. bone marrow cell populations in control rats and those exposed to the laser driven UPEB irradiation.

Cells	Control (M ± SD)	Anesthesia (M ± SD)	1st day (M ± SD)	3rd day (M ± SD)	7th day (M ± SD)	14th day (M ± SD)	28th day (M ± SD)
Proerythroblast	1.4 ± 0.7	1.8 ± 1.1	2.7 ± 1.1	1.0 ± 0.6	0.7 ± 0.2	0.8 ± 0.1	1.0 ± 0.6
Basophil erytroblasts	14.1 ± 4.0	15.7 ± 3.2	8.1 ± 3.1	16.2 ± 5.2	16.1 ± 1.5	16.9 ± 8.3	13.0 ± 4.1
Ortochrom erytroblasts	10.1 ± 5.8	11.1 ± 6.0	11.8 ± 3.3	16.4 ± 7.3	11.0 ± 2.4	18.5 ± 4.5	11.0 ± 2.8
Lymphoblast	5.1 ± 0.8	4.4 ± 1.1	3.1 ± 1.5	4.1 ± 1.7	3.8 ± 1.1	3.3 ± 1.3	4.1 ± 2.3
Lymphocytes	29.9 ± 8.2	26.0 ± 8.9	12.7 ± 2.6*	22.9 ± 5.6	26.3 ± 10.0	23.7 ± 4.2	25.8 ± 4.8
Monoblast	3.4 ± 1.0	3.5 ± 1.7	2.5 ± 0.8	2.3 ± 1.0	1.7 ± 0.6*	1.5 ± 0.6*	1.2 ± 0.6*
Monocyte	1.6 ± 1.1	2.6 ± 1.1	3.1 ± 0.9	2.9 ± 2.2	2.1 ± 0.3	2.0 ± 0.4	2.0 ± 0.6
Myeloid	4.1 ± 2.7	3.3 ± 1.6	4.2 ± 1.3	2.9 ± 0.9	3.2 ± 0.7	3.6 ± 1.0	2.9 ± 0.8
Metamielocyte	4.7 ± 2.4	4.8 ± 1.1	6.0 ± 2.5**	4.4 ± 1.2	3.5 ± 0.9	3.6 ± 1.6	3.2 ± 0.3
Band	7.4 ± 2.5	9.9 ± 2.7	16.7 ± 6.4**	8.6 ± 4.0	8.6 ± 4.7	5.7 ± 3.4	11.5 ± 1.1**
Segment	7.6 ± 3.2	9.1 ± 2.6	12.9 ± 3.9**	3.2 ± 0.9*	2.8 ± 1.8*	2.0 ± 1.1*	4.1 ± 0.7*
Pathological neutrophils	-	-	4.6 ± 2.0**	3.1 ± 2.0**	5.9 ± 3.0**	3.4 ± 1.1**	3.2 ± 1.1**
Eosinophil	9.5 ± 4.8	7.1 ± 1.2	7.1 ± 4.1	4.5 ± 3.1	1.3 ± 0.9	3.9 ± 3.1	7.9 ± 7.6
Basophil	0.4 ± 0.5	0.4 ± 0.2	0.6 ± 0.6	0.2 ± 0.4	0.2 ± 0.2	0.2 ± 0.2	0.3 ± 0.2
Megakaryocyte	0.3 ± 0.4	0.3 ± 0.1	0.6 ± 0.4	0.3 ± 0.2	0.2 ± 0.2	0.5 ± 0.1	0.5 ± 0.3
Macrophage	0.3 ± 0.4	0.2 ± 0.3	0.5 ± 0.5	1.1 ± 0.9	0.4 ± 0.4	0.6 ± 0.4	0.7 ± 0.3
Pathological lymphocytes	-	0.1 ± 0.2	0.3 ± 0.3	0.5 ± 0.2**	1.9 ± 1.0**	1.1 ± 0.2**	1.0 ± 0.5**
Destroyed	-	-	2.6 ± 1.9**	5.2 ± 2.1**	9.1 ± 4.9**	8.2 ± 4.6**	6.2 ± 2.7**
Pathological erythrocytes	-	-	-	0.5 ± 0.4**	1.2 ± 0.8**	0.9 ± 0.6**	0.5 ± 0.4**

Legend: *significantly lower compared with controls ($p < 0.05 – p < 0.01$); ** significantly higher compared with controls ($p < 0.05 – p < 0.01$).

Table S3. lymph node cell populations in control rats and those exposed to the laser driven UPEB irradiation.

Percentage	Control (M ± SD)	Anesthesia (M ± SD)	1st day (M ± SD)	3rd day (M ± SD)	7th day (M ± SD)	14th day (M ± SD)	28th day (M ± SD)
Proerythroblast	0.4 ± 0.2	0.2 ± 0.1	0.1 ± 0.1	0.1 ± 0.1	0.2 ± 0.1	0.03 ± 0.03	0.01 ± 0.1
Basophil erythroblasts	0.6 ± 0.2	1.5 ± 0.6	0.5 ± 0.7	2.0 ± 0.3**	2.5 ± 0.6**	0.7 ± 0.4	0.6 ± 0.4
Ortochrom erythroblasts	2.8 ± 0.9	2.4 ± 0.4	2.3 ± 1.7	0.9 ± 0.4*	2.1 ± 1.8	1.6 ± 1.6	1.4 ± 0.7
Lymphoblast	2.4 ± 0.8	3.6 ± 0.9	6.7 ± 1.9**	7.0 ± 1.9**	4.1 ± 0.8	1.5 ± 0.08	3.4 ± 0.3
Lymphocytes	88.3 ± 2.8	89.6 ± 1.0	40.1 ± 4.7*	66.7 ± 7.0*	81.2 ± 3.3	81.4 ± 0.03	82.7 ± 6.1
Monoblast	1.3 ± 0.7	0.8 ± 0.2	1.0 ± 0.2	0.7 ± 0.1	0.2 ± 0.1*	0.5 ± 0.4	0.1 ± 0.05*
Monocyte	0.6 ± 0.4	0.3 ± 0.2	0.5 ± 0.3	0.5 ± 0.3	0.2 ± 0.1	0.2 ± 0.21	0.05 ± 0.05*
Myeloid	0.7 ± 0.5	1.0 ± 0.4	0.5 ± 0.2	1.6 ± 0.2	0.3 ± 0.2	0.5 ± 0.2	0.11 ± 0.6*
Metamielocyte	0.6 ± 0.1	0.1 ± 0.1	0.1 ± 0.1	0.2 ± 0.2	0.3 ± 0.1	0.5 ± 0.03	0.4 ± 0.4
Band	0.6 ± 0.3	0.1 ± 0.1	0.5 ± 0.3	0.7 ± 0.4	0.1 ± 0.1	0.6 ± 0.03	0.2 ± 0.2
Segment	0.5 ± 0.2	0.1 ± 0.1	0.6 ± 0.4	1.,4 ± 0.7**	0.3 ± 0.06	0.6 ± 0.4	0.2 ± 0.2
Pathol neutrophil	0.0	0.0	0.9 ± 0.4**	1.5 ± 0.9**	0.9 ± 0.4**	1.8 ± 0.2**	0.5 ± 0.3**
Pathol. lymphocyte	0.0	0.0	0.4 ± 0.3	0.3 ± 0.2	0.7 ± 0.2	0.5 ± 0.3	0.6 ± 0.3
Eosinophil	0.8 ± 0.4	0.1 ± 0.1	0.3 ± 0.1	1.4 ± 0.8	0.2 ± 0.1	0.1 ± 0.01	1.0 ± 0.9
Basophil	0.1 ± 0.1	0.01 ± 0.01	0.04 ± 0.04	0.3 ± 0.2	0.06 ± 0.05	0.03 ± 0.03	0.0
Megakaryocyte	0.0	0.0	0.02 ± 0.03	0.0	0.0	0.0	0.0
Macrophages	0.4 ± 0.2	0.2 ± 0.1	0.4 ± 0.3	0.2 ± 0.2	0.1 ± 0.1	0.0*	0.4 ± 0.4
Destroyed	0.0	0.0	14.2 ± 2.5**	14.4 ± 4.2**	4.6 ± 1.3**	9.5 ± 1.8**	8.4 ± 4.4**
Cells per field	46.7 ± 5.3	57.8 ± 4.8	21.9 ± 3.1*	22.1 ± 1.3*	36.3 ± 2.7*	32 ± 4.4*	36.6 ± 6.0

Legend: *significantly lower compared with controls or tendency ($p < 0.1 – p < 0.01$); **significantly higher compared with controls or tendency ($p < 0.1 – p < 0.01$).
